# Supplementary material for: Persistent COVID-19 symptoms in community-living older adults from the Canadian Longitudinal Study on Aging (CLSA)
Source: Commun Med (Lond). 2023 Mar 11;3:36. doi: 10.1038/s43856-023-00266-0 (PMC10006564; doi:10.1038/s43856-023-00266-0)
Supplement: Supplementary file 1 — Description of Additional Supplementary Files [file 43856_2023_266_MOESM1_ESM.pdf]

Description of Additional Supplementary Files

File Name: Supplementary Data 1

Description: Data to reproduce Figure 1

File Name: Supplementary Data 2

Description: Data to reproduce Figures 2a and 2b

File Name: Supplementary Data 3

Description: Data to reproduce Figure 3
