# Supplementary material for: Persistent COVID-19 symptoms in community-living older adults from the Canadian Longitudinal Study on Aging (CLSA)
Source: Commun Med (Lond). 2023 Mar 11;3:36. doi: 10.1038/s43856-023-00266-0 (PMC10006564; doi:10.1038/s43856-023-00266-0)
Supplement: Supplementary file 2 — Supplementary Information [file 43856_2023_266_MOESM2_ESM.pdf]

Supplementary Information

Persistent COVID-19 symptoms in community-living older adults from the Canadian Longitudinal Study on Aging (CLSA)

Lauren E. Griffith, PhD[1,2,3]
Marla Beauchamp, PhD[4,2,3]
Jacqueline McMillan, MD MSc[5]
Sayem Borhan, PhD[1]
Urun Erbas Oz, PhD[1]
Christina Wolfson, PhD[6,7,8]
Susan Kirkland, PhD[9]
Nicole E. Basta, PhD[6]
Mary Thompson, PhD[10]
Parminder Raina, PhD[1,2,3]
on behalf of the Canadian Longitudinal Study on Aging (CLSA) Team*

Affiliations:
[1]Department of Health Research Methods, Evidence, and Impact, McMaster University, Hamilton, Canada
[2]Labarge Centre for Mobility in Aging, McMaster University, Hamilton, Ontario, Canada
[3]McMaster Institute for Research on Aging, McMaster University, Hamilton, Ontario, Canada
[4]School of Rehabilitation Science, McMaster University, Hamilton, Ontario, Canada
[5]Division of Geriatric Medicine, Department of Medicine, Cumming School of Medicine, University of Calgary, Calgary, AB, Canada
[6]Department of Epidemiology, Biostatistics, and Occupational Health, McGill University, Montreal, Quebec, Canada
[7]Department of Medicine, McGill University, Montreal, Quebec, Canada
[8]Research Institute of the McGill University Health Centre, Quebec, Canada
[9]Department of Community Health & Epidemiology, Dalhousie University, Halifax, Nova Scotia, Canada
[10]Department of Statistics and Actuarial Science, University of Waterloo, Waterloo, Ontario, Canada.
* A list of authors and their affiliations appears at the end of the paper.

Supplementary Figure 1. Cumulative incidence (percent) of mild (dark blue) and moderate/severe (light blue) symptoms since March 2020 reported by CLSA COVID-19 study participant who had COVID-19 (n=121) and who did not have COVID-19 (n=23,636). The COVID-19 group is standardized to reflect the age-, sex- and multimorbidity-distribution of the non-COVID-19 group

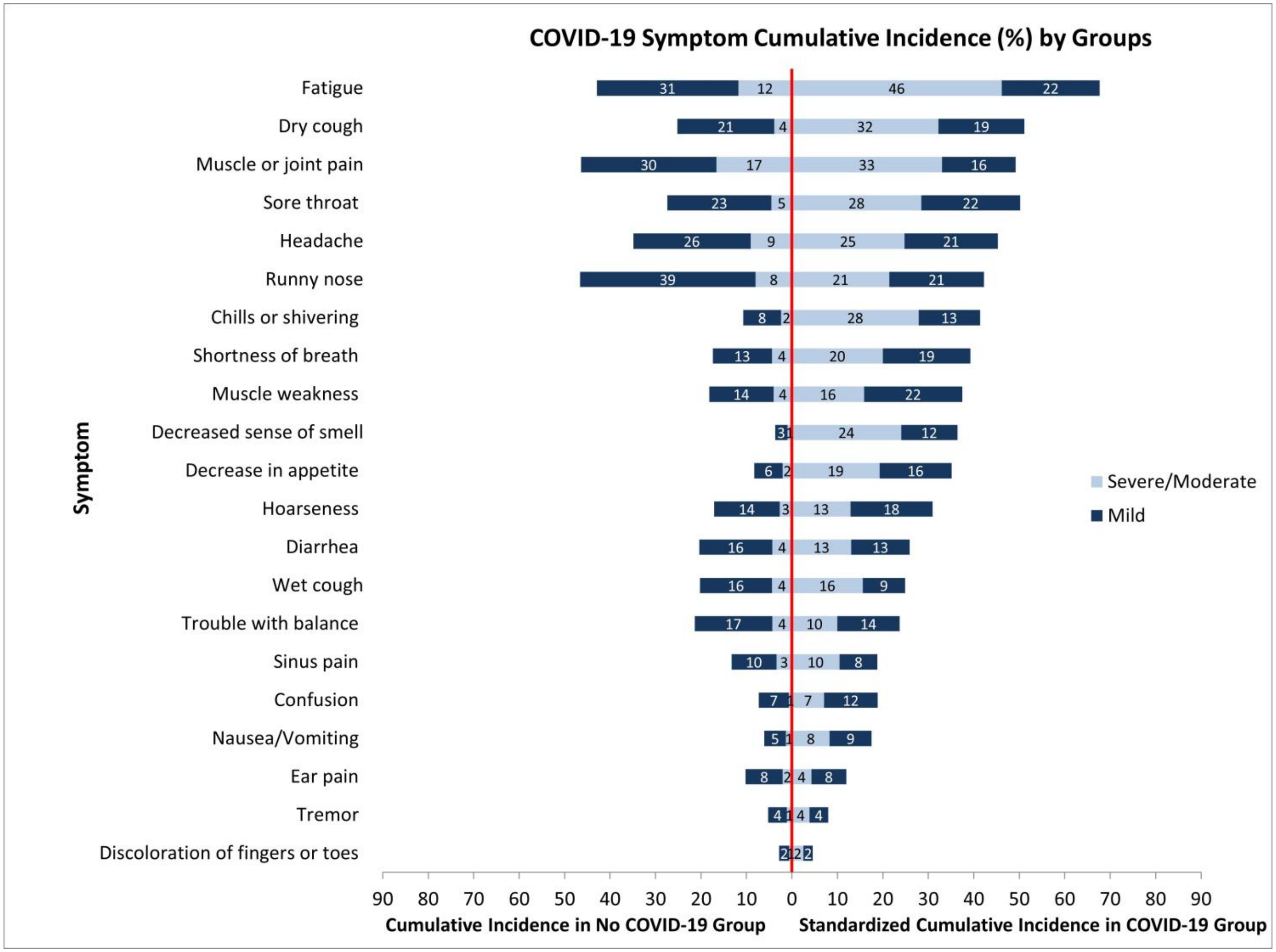

Supplementary Figure 2a-c. Cumulative Incidence percent of mild (dark blue) and moderate/severe (light blue) symptoms since March 2020 reported by non-COVID-19 and COVID-19 participant age <65 (2a), 65-74 (2b) and 75+ (2c) years

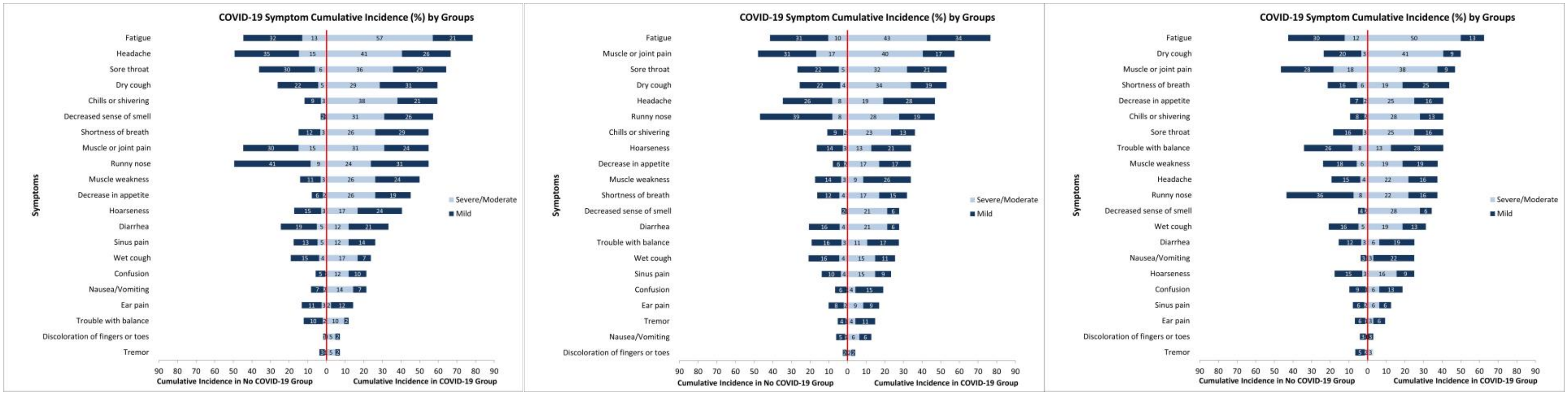

Supplementary Figure 3a-b Number of Persistent Symptoms >1 month (3a) and >3 months (3b) in Female and Male Participants with a Positive COVID-19 Test or Physician Diagnosis

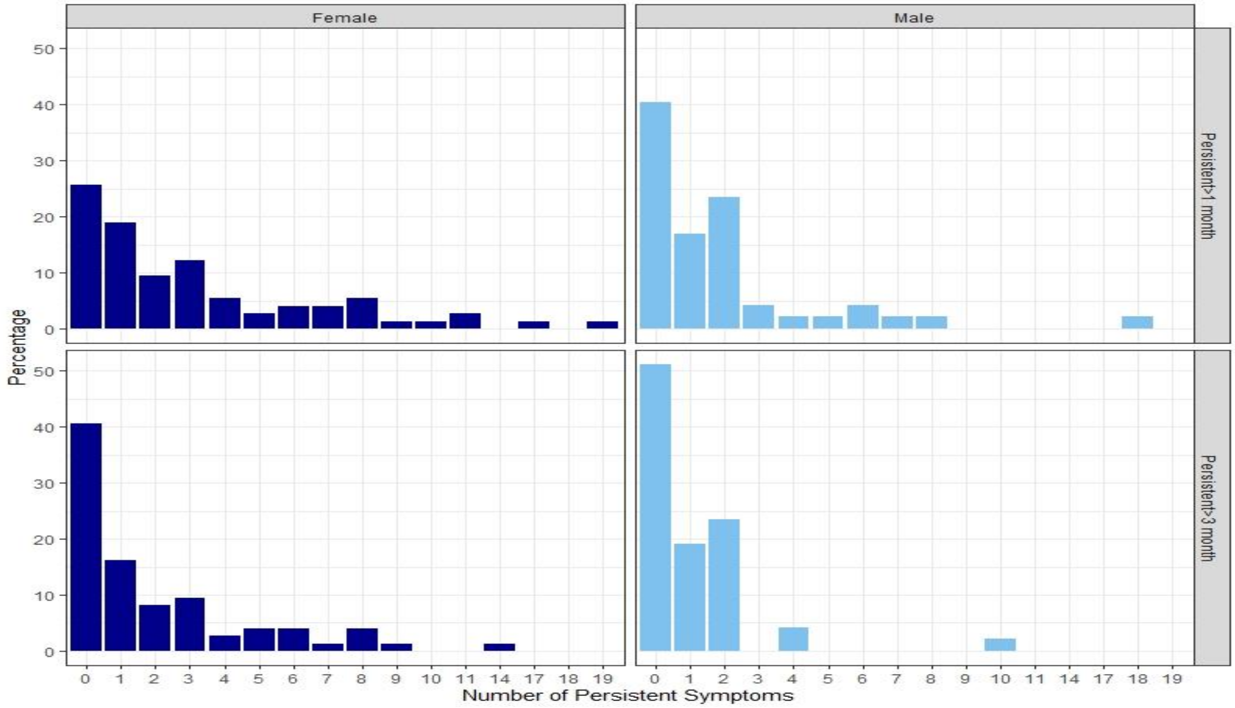

Supplementary Figure 4a-b Number of Persistent Symptoms >1 month (4a) and >3 months (4b) in Participants Aged <65, 65-74 and 75+ Years with a Positive COVID-19 Test or Physician Diagnosis

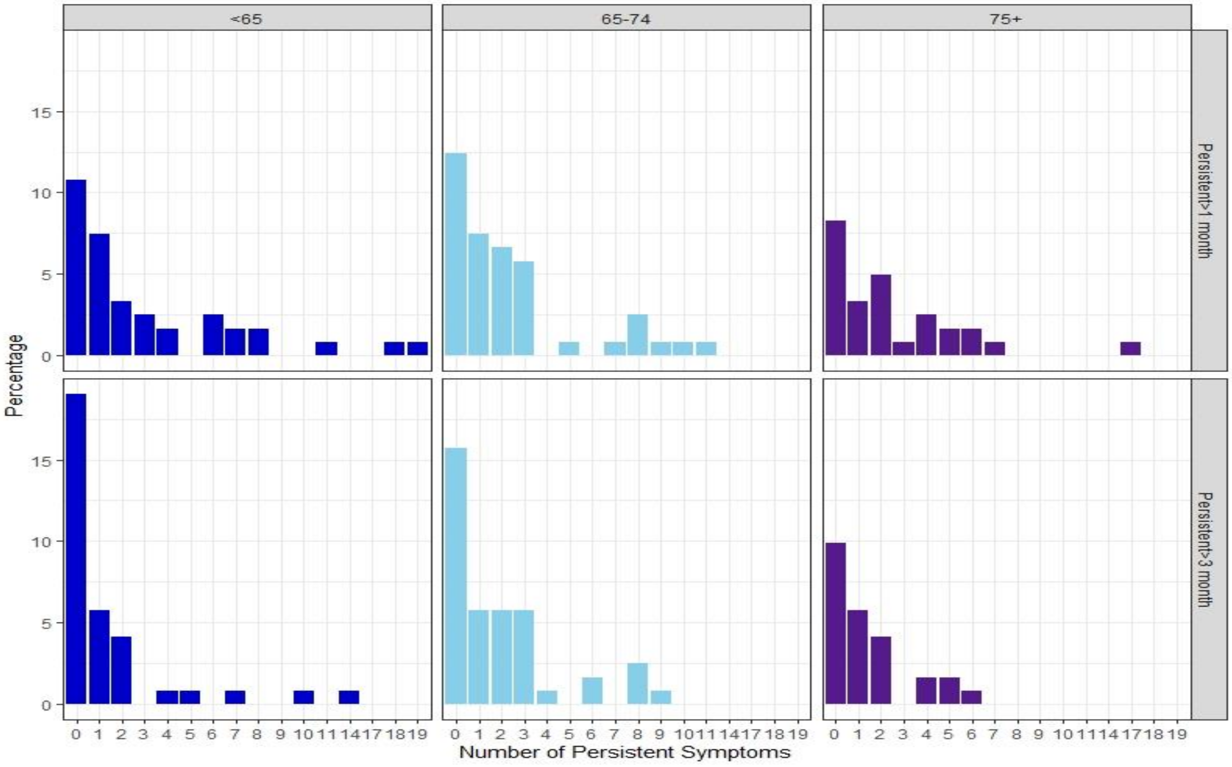

Supplementary Table 1. List of Research Ethics Boards for the Canadian Longitudinal Study on Aging

Institution	Province	REB
University of Victoria	British Columbia	Human Research Ethics Office of Research Services
Vancouver Island Health Authority (VIHA)	British Columbia	Island Health Research Ethics Board
University of British Columbia	British Columbia	University of British Columbia Clinical Research Ethics Board
Simon Fraser University	British Columbia	Simon Fraser University Office of Research Ethics
University of Calgary	Alberta	Conjoint Health Research Ethics Board (CHREB)
University of Manitoba	Manitoba	Bannatyne Campus Health Research Ethics Board (HREB)
McMaster University	Ontario	Hamilton Integrated Research Ethics Board (HiREB)
Elisabeth Bruyere Research Institute of Ottawa	Ontario	Bruyère Continuing Care Research Ethics Board
McGill University	Quebec	McGill Institutional Review Board
Other McGill REB	Quebec	Research Institute of the McGill University Health Centre
Universite de Sherbrooke	Quebec	Centre de santé et des services sociaux- Institut universitaire de gériatrie de Sherbrooke (?)
Dalhousie University	Nova Scotia	Office of Research Ethics Administration
PEI Health	Prince Edward Island	PEI Research Ethics Board
Memorial University of Newfoundland	Newfoundland and Labrador	Faculty of Medicine Health Research Ethics Authority

Supplementary Table 2a. Comparison of sociodemographic and health characteristics of the CLSA Baseline cohort and participants completing the COVID-19 Exit questionnaire

	BASELINE (n=51,338)		CLSA Follow-up 1 (n=44,817)		COVID-19 EXIT (n=24,114)	
	n	%	n	%	n	%
**Age Group**[1]						
<55	13427	26.15	6598	14.72	890	3.69
55-64	16420	31.98	14751	32.91	7136	29.59
65-74	11996	23.37	13302	29.68	8856	36.73
75+	9495	18.5	10166	22.68	7232	29.99
**Sex**						
Female	26155	50.95	22944	51.19	12819	53.16
Male	25183	49.05	21873	48.81	11295	46.84
**Ethnicity**						
European	47105	92.68	41273	92.95	22439	93.79
Non-European	3718	7.32	3132	7.05	1485	6.21
**Annual Household Income**						
Less than $20,000	2913	6.07	2083	5.01	861	3.80
$20,000-$49,999	12209	25.42	9929	23.89	4855	21.45
$50,000-$99,999	17127	35.66	15124	36.39	8571	37.87
$100,000-$149,999	8739	18.20	7810	18.79	4589	20.27
$150,000 or more	7039	14.66	6616	15.92	3758	16.60
**Living Situation**						
Living alone	11747	22.89	10704	24.35	5991	25.33
Not living alone	39564	77.11	33261	75.65	17663	74.67
**Living Area**						
Rural	9634	18.77	6660	14.87	4278	17.84
Urban	41704	81.23	38126	85.13	19706	82.16
**Number of Chronic Conditions**						
0	10288	20.86	5246	12.18	2794	12.06
1	12733	25.81	8560	19.87	4775	20.61
2	10686	21.66	8970	20.82	5064	21.86
3+	15618	31.66	20308	47.14	10532	45.47
**Type of Alcohol Drinker**						
Non-drinkers during last 12 months	7644	14.90	6007	13.42	4350	18.18
Binge drinker	2691	5.24	1996	4.46	1818	7.60
Regular/Occasional drinker	40974	79.81	36752	82.12	17760	73.65
**Type of Smoker**						
Current smoker	4843	9.49	3285	7.37	1448	6.11
Former smoker	30530	59.80	27313	61.3	14729	62.15
Never smoked	15684	30.72	13961	31.33	7523	31.74

[1]An average of 7 years passed between CLSA baseline and the COVID-19 exit interview

Supplementary Table 2b. Comparison of sociodemographic and health characteristics from CLSA Follow-up1 for participants who completed the COVID-19 Exit questionnaire (n=24,114) and participants who did not complete the COVID-19 Exit questionnaire (n=18,343)

	Completed COVID-19 EXIT (n=24,114)		Did not Complete COVID-19 Exit Survey (n=18,343)		Standardized Mean Difference
**Age Group**	n	%	n	%	
<55	3,155	13.16	3,259	18.20	-0.139
55-64	8,314	34.68	5,919	33.06	0.034
65-74	7,804	32.55	4,678	26.13	0.141
75+	4,701	19.61	4,047	22.61	-0.073
**Sex**					
Female	12,819	53.16	8,960	48.85	0.086
Male	11,295	46.84	9,383	51.15	-0.086
**Ethnicity**					
European	22,439	93.79	16,612	91.58	0.085
Non-European	1,485	6.21	1,527	8.42	-0.085
**Annual Household Income**					
Less than $20,000	861	3.80	988	5.98	-0.101
$20,000-$49,999	4,855	21.45	4,223	25.56	-0.097
$50,000-$99,999	8,571	37.87	5,702	34.51	0.070
$100,000-$149,999	4,589	20.27	2,933	17.75	0.075
$150,000 or more	3,758	16.6	2,676	16.2	0.011
**Living Situation**					
Living alone	5,599	23.41	4,261	24.04	-0.015
Not living alone	18,323	76.59	13,460	75.96	0.015
**Living Area**					
Rural	3,309	13.81	2,926	16.36	-0.071
Urban	20,649	86.19	14,964	83.64	0.071
**Number of Chronic Conditions**					
0	2,794	12.06	2,318	13.41	-0.041
1	4,775	20.61	3,468	20.07	0.014
2	5,064	21.86	3,524	20.39	0.036
3+	10,532	45.47	7,970	46.12	-0.013
**Type of Alcohol Drinker**					
Non-drinkers during last 12 mo.	2,777	11.6	2,586	14.47	-0.085
Binge drinker	968	4.04	926	5.18	-0.054
Regular/Occasional drinker	20,202	84.36	14,364	80.35	0.105
**Type of Smoker**					
Current smoker	1,387	5.82%	1837	8.81	-0.119
Former smoker	14,787	62.05%	12584	60.33	0.041
Never smoked	7,656	32.13%	6438	30.86	0.024

Supplementary Table 3. Average number of questionnaires (0-6) on which a symptom was reported by persistence status (>1 month) at exit survey (n=121)

**Symptom**[1]	Not Persistent >1 m		Persistent >1 m		P-value
	n	mean (SD)	n	mean (SD)	
Fatigue	81	1.75 (1.79)	38	3.87 (1.96)	<0.001
Dry cough	94	1.09 (1.34)	25	3.20 (2.24)	<0.001
Muscle or joint pain	96	1.69 (1.70)	23	3.57 (1.97)	<0.001
Sore throat	104	1.06 (1.32)	12	2.75 (2.09)	<0.001
Headache	106	1.37 (1.63)	11	3.00 (2.19)	0.003
Runny nose	96	1.43 (1.48)	21	3.57 (1.57)	<0.001
Chills or shivering	115	0.56 (0.85)	5	2.60 (2.41)	<0.001
Shortness of breath	94	0.65 (1.37)	26	3.04 (1.75)	<0.001
Decreased sense of smell	97	0.47 (1.04)	21	2.19 (2.25)	<0.001
Decrease in appetite	106	0.63 (0.82)	13	1.92 (1.93)	<0.001
Hoarseness	104	0.93 (1.61)	7	3.71 (1.98)	<0.001
Diarrhea	112	0.75 (1.30)	8	4.62 (1.69)	<0.001
Wet cough	103	0.56 (1.19)	15	3.80 (1.86)	<0.001
Sinus pain	114	0.53 (1.07)	6	1.50 (2.51)	0.048
Confusion	110	0.25 (0.78)	8	3.38 (2.13)	<0.001
Nausea/Vomiting	118	0.38 (0.65)	3	4.33 (2.89)	<0.001
Ear pain	114	0.51 (1.03)	5	1.80 (1.10)	0.007

[1] Muscle weakness, tremor, trouble with balance, and discoloration of fingers and toes were not included because they were added after the baseline questionnaire

Supplementary Table 4. Other Symptoms Reported Beyond the Prespecified List of 23 by Participants with a Positive COVID-19 Test or Physician Diagnosis of COVID-19

**Symptom Category**	**Symptom Description**
Cardiac	Cardiac ischemia
Cardiac	Stiffening of heart muscle
Cardiac	CAS - Coronary Arterial Spasms, Heart Attack
Cardiac	Hypertension
Cognitive	Short term memory seems to be weak.
Dermatologic	Skin rash on back
Dermatologic	Red skin spots
Lower Respiratory	Minor pneumothoraxes - bleb releases, increase in lung cysts
Neurological	Continuous Tingling in arms and legs for 10 weeks then intermittently for 4 months gradually disappearing
Neurological	Pain and tingling on edge of feet
Neurological	Leg numbness
Neurological	prickly skin sensation on fore arms (like a rash)
Other	feeling of burning in the thorax

Supplementary Table 5. Detailed Step-wise Results for Multivariable Negative Binomial Regression Analysis Assessing Factors Associated with >1 Month and >3 Month Symptom Persistence in Participants with a Positive COVID-19 Test or Physician Diagnosis

**Factors**	**AIC**	**Factors**	**AIC**
**Outcome: persistent symptoms >1 month**		**Outcome: persistent symptoms >3 month**	
**Step 1**		**Step 1**	
Age Group (ref: <65 years) + Sex (ref: Female)	474.30	Age Group (ref: <65 years) + Sex (ref: Female)	369.88
+Number of Chronic Conditions (ref:0 or 1)	473.93	+Number of Chronic Conditions (ref:0 or 1)	369.03
+Any Depression or Anxiety (ref: No)	475.43	+Any Depression or Anxiety (ref: No)	371.78
+Any Mobility ADL difficulty (ref: No)	476.18	+Any Mobility ADL difficulty (ref: No)	369.26
+Subjective social status	476.30	+Subjective social status	368.61
**Step 2**		**Step 2**	
Age Group (ref: <65 years) + Sex (ref: Female) + Number of Chronic Conditions (ref:0 or 1)	473.93	Age Group (ref: <65 years) + Sex (ref: Female) + Subjective social status	368.61
+Any Depression or Anxiety (ref: No)	474.80	+ Number of Chronic Conditions (ref:0 or 1)	367.50
+Any Mobility ADL difficulty (ref: No)	475.84	+Any Mobility ADL difficulty (ref: No)	369.01
+Subjective social status	475.92	+Any Depression or Anxiety (ref: No)	370.60
-Number of Chronic Conditions (ref:0 or 1)	474.30	-Subjective social status	369.88
		**Step 3**	
		Age Group (ref: <65 years) + Sex (ref: Female) + Subjective social status+ Number of Chronic Conditions (ref:0 or 1)	367.50
		- Number of Chronic Conditions (ref:0 or 1)	368.61
		-Subjective social status	369.03
		+Any Mobility ADL difficulty (ref: No)	368.32
		+Any Depression or Anxiety (ref: No)	369.48

Supplementary Table 6. Multivariable Negative Binomial Regression Analysis Results Assessing Factors Associated with >1 Month and >3 Month Symptom Persistence in Participants with a Positive COVID-19 Test or Physician Diagnosis using 5 Imputation Datasets

**Factors**	**Any persistent symptoms >1 month**		**Any persistent symptoms >3 month**	
	**aIRR (95% CI)**	**p-value**	**aIRR (95% CI)**	**p-value**
Age Group (ref: <65 years)				
65-74	0.69 (0.40, 1.18)	0.151	1.23 (0.65, 2.32)	0.713
75+	0.65 (0.35, 1.21)		0.83 (0.40, 1.72)	
Female Sex	1.64 (1.01, 2.68)	0.048	1.43 (0.81, 2.54)	0.220
Number of Chronic Conditions (ref:0 or 1)				
2 or more	1.85 (0.99, 3.44)	0.051	2.15 (1.04, 4.45)	0.039
Any Depression or Anxiety (ref: No)				
Subjective social status (1 rung)			0.85 (0.75, 0.98)	0.025
Any Mobility ADL difficulty (ref: No)				

[1]COVID-19 Status based on self-reported positive COVID-19 test, physician diagnosis.

Supplementary Table 7. Multivariable Negative Binomial Regression Analysis Results Assessing Factors Associated with >1 Month and >3 Month Symptom Persistence in Participants with a Positive COVID-19 Test or Physician Diagnosis (n=121) OR who indicating they "very likely" had COVID-19 and had seroprevalence results indicating that they had COVID-19 antibodies and were not vaccinated (n=15)

**Factors**	**Univariate**		**Multivariable**	
	**IRR (95% CI)**	**p-value**	**IRR (95% CI)**	**p-value**
Outcome: Any persistent symptoms >1 month				
Age Group (ref: <65 years)				
65-74	0.78 (0.46, 1.33)	0.655	0.68 (0.40, 1.13)	0.295
75+	0.87 (0.48, 1.58)		0.71 (0.39, 1.31)	
Female Sex	1.61 (1.01, 2.56)	0.043	1.61 (1.01, 2.54)	0.042
Number of Chronic Conditions (ref:0 or 1)				
2 or more	2.00 (1.12, 3.50)	0.016	2.13 (1.18, 3.80)	0.011
Any Depression or Anxiety (ref: No)	0.98 (0.61, 1.62)	0.950		
Subjective social status (1 rung)	0.96 (0.85, 1.07)	0.468		
Any Mobility ADL difficulty (ref: No)	1.39 (0.83, 2.43)	0.227		
Outcome: Any persistent symptoms >3 months				
Age Group (ref: <65 years)				
65-74	1.22 (0.66, 2.26)	0.785	1.01 (0.56, 1.83)	0.909
75+	1.03 (0.51, 2.10)		0.88 (0.43, 1.79)	
Female Sex	1.87 (1.09, 3.20)	0.022	1.41 (0.80, 2.47)	0.214
Number of Chronic Conditions (ref:0 or 1)				
2 or more	2.28 (1.15, 4.44)	0.016	2.13 (1.07, 4.23)	0.033
Any Depression or Anxiety (ref: No)	1.29 (0.74, 2.28)	0.372		
Subjective social status (1 rung)	0.84 (0.73, 0.96)	0.008	0.87 (0.75, 0.99)	0.040
Any Mobility ADL difficulty (ref: No)	1.89 (1.06, 3.05)	0.035		

Rationale for use of seroprevalence results as a sensitivity analysis: A seroprevalence study in the CLSA was conducted between November 12, 2020-August 31, 2021. There are a number of reasons that we chose not to use seroprevalence information to characterize our population in this study as COVID-19 positive. The self-reported COVID-19 status and persistent symptom data were collected from the beginning of the pandemic until COVID-19 exit survey completion (September-December 2020). While some people with COVID-19 would still have antibodies at the time of the seroprevalence study, it has been shown that the antibodies decrease significantly by 4 months post infection (DOI: 10.1126/sciimmunol.abe0367) and thus there would be many false negatives. As well a definitive "COVID-19 infection" positive was not possible in people who were vaccinated. In Canada, over 90% of the population 50 years and older had their first vaccination by mid-May. Although we acknowledge that there will be some misclassification with self-reported COVID-19 status, we feel that because a positive COVID-19 test or physician diagnosis was such a salient event in the first year of the pandemic that recall bias will be minimal.

As a sensitivity analysis used the seroprevalence results to increase our sample size for our persistent symptom regression analysis by including the subgroup of participants who said it was very likely that they had COVID but did not report a positive test or physician diagnosis (excluded from our primary analyses) who also had a positive seroprevalence results and were not yet vaccinated.
