# Supplementary material for: Persistent COVID-19 symptoms in community-living older adults from the Canadian Longitudinal Study on Aging (CLSA)
Source: Commun Med (Lond). 2023 Mar 11;3:36. doi: 10.1038/s43856-023-00266-0 (PMC10006564; doi:10.1038/s43856-023-00266-0)
Supplement: Supplementary file 7 — Reporting Summary [file 43856_2023_266_MOESM7_ESM.pdf]

nature portfolio

Corresponding author(s): Parminder Raina

Last updated by author(s): Feb 8, 2023

Reporting Summary

Nature Portfolio wishes to improve the reproducibility of the work that we publish. This form provides structure for consistency and transparency in reporting. For further information on Nature Portfolio policies, see our Editorial Policies and the Editorial Policy Checklist.

Statistics

For all statistical analyses, confirm that the following items are present in the figure legend, table legend, main text, or Methods section.

n/a	Confirmed	
☐	☒	The exact sample size (n) for each experimental group/condition, given as a discrete number and unit of measurement
☐	☒	A statement on whether measurements were taken from distinct samples or whether the same sample was measured repeatedly
☐	☒	The statistical test(s) used AND whether they are one- or two-sided<br>*Only common tests should be described solely by name; describe more complex techniques in the Methods section.*
☐	☒	A description of all covariates tested
☐	☒	A description of any assumptions or corrections, such as tests of normality and adjustment for multiple comparisons
☐	☒	A full description of the statistical parameters including central tendency (e.g. means) or other basic estimates (e.g. regression coefficient) AND variation (e.g. standard deviation) or associated estimates of uncertainty (e.g. confidence intervals)
☐	☒	For null hypothesis testing, the test statistic (e.g. F, t, r) with confidence intervals, effect sizes, degrees of freedom and P value noted<br>*Give P values as exact values whenever suitable.*
☒	☐	For Bayesian analysis, information on the choice of priors and Markov chain Monte Carlo settings
☒	☐	For hierarchical and complex designs, identification of the appropriate level for tests and full reporting of outcomes
☐	☒	Estimates of effect sizes (e.g. Cohen's d, Pearson's r), indicating how they were calculated

Our web collection on statistics for biologists contains articles on many of the points above.

Software and code

Policy information about availability of computer code

Data collection	CLSA has developed core suite of software based on open source code to collect data. The specific software used to collect data include ONYX (version 1.12.0), Limesurvey (version 3.7.1 with customizations), and PINE (version 2.7). www.clsa-elcv.ca
Data analysis	Statistical analysis was conducted using R version 4.1.0. Custom code that supports the results of this study can be made available upon request from the corresponding author.

For manuscripts utilizing custom algorithms or software that are central to the research but not yet described in published literature, software must be made available to editors and reviewers. We strongly encourage code deposition in a community repository (e.g. GitHub). See the Nature Portfolio guidelines for submitting code & software for further information.

Data

Policy information about availability of data

All manuscripts must include a data availability statement. This statement should provide the following information, where applicable:

- Accession codes, unique identifiers, or web links for publicly available datasets
- A description of any restrictions on data availability
- For clinical datasets or third party data, please ensure that the statement adheres to our policy

Data are available from the Canadian Longitudinal Study on Aging (www.clsa-elcv.ca) for researchers who meet the criteria for access to de-identified CLSA data.

Field-specific reporting

Please select the one below that is the best fit for your research. If you are not sure, read the appropriate sections before making your selection.

☐ Life sciences ☒ Behavioural & social sciences ☐ Ecological, evolutionary & environmental sciences

For a reference copy of the document with all sections, see nature.com/documents/nr-reporting-summary-flat.pdf

Behavioural & social sciences study design

All studies must disclose on these points even when the disclosure is negative.

Study description	The current study uses data from a cross-sectional COVID-19 questionnaire administered online or by telephone from September-December 2020 linked to baseline (2011-2015) and follow-up 1 (2015-2018) data from a pre-existing population-based cohort, the Canadian Longitudinal Study on aging (CLSA). The CLSA recruited 51,388 community-dwelling adults from the 10 Canadian provinces at baseline (2011-2015) to be followed every 3 years, for at least 20 years or until death, to collect information on the changing biological, medical, psychological, social, lifestyle and economic aspects of people's lives. Of these participants, 28,559 were recruited to participate in the CLSA COVID-19 Study and 24,114 completed the Exit questionnaire. We report data from the Exit interview and use pre-COVID-19 data from CLSA baseline and follow-up 1 as predictors of COVID-19 symptom persistence.
Research sample	The Canadian Longitudinal Study on Aging (CLSA) is a large, national, longitudinal cohort study that recruited 51,338 Canadian residents aged 45-85 years at baseline (2011-2015). The middle-aged adults were included at baseline to capture mid-life experiences prospectively, since many important transitions that impact health outcomes later in life tend to occur during this period. Adults as old as 85 years were included to prospectively examine transitions into the final years of life.<br>The first follow-up was completed on 48,893 participants (95% retention) in mid-2018. A total of 42,511 participants from the core CLSA study were eligible to participate in the CLSA COVID-19 study of which 28,559 (67.2) agreed to participate and 24,114 (56.7%) completed the COVID-19 exit interview. Of the total participants recruited at CLSA baseline, 50.95% were females, 41.87% were aged 65 years and older, 92.68% were of European ethnic background, and 68.52% had total household income of $50,000 or more.
Sampling strategy	A stratified, random sampling was used to sample participants into the core CLSA study. Two methods were used to recruit participants into the CLSA COVID-19 Study: via e-mail and by telephone. To calculate the CLSA sample size at baseline, the expected number of outcomes were estimated for each 3-year follow-up period based on age and sex specific incidence rates and after accounting for mortality and loss-to-follow-up. In the next step, the expected number of outcomes and a range of exposure/risk factor prevalence (5%, 10%, 20%, and 50%) were used to estimate the minimum detectable odds ratio between an exposure and outcome, after accounting for misclassification of the exposure and outcome. This iterative simulation-based approach demonstrated that the CLSA data is sufficiently powered to examine a wide range of associations, and especially those involving more common outcomes such as depressive symptoms.
Data collection	The CLSA recruited 51,338 Canadian residents aged 45-85 years at baseline (2011-2015), to be followed every three years, for at least 20 years or until death or loss to follow-up, to collect information on the changing biological, medical, psychological, social, lifestyle and economic aspects of people's lives. At each study visit, all participants provide data by questionnaires but a subset of 30,097 participants living within 25-50 km of one of 11 Data Collection Sites (DCS) across seven Canadian provinces are also interviewed in their home and visit a DCS to provide a range of physical assessments and biological samples. The first follow-up was completed on 48,893 participants in mid-2018.<br>The CLSA COVID-19 questionnaire-based study was launched on April 15, 2020 to understand the epidemiology of COVID-19 including social and mental health consequences of the pandemic among older Canadians. Participants who had active email addresses were contacted by email and invited to participate via a web questionnaires (n=34,498) and those without an active email address were invited to complete the interviews by telephone (n=8,202) where information was entered for them by trained CLSA interviewers.
Timing	The CLSA baseline data were collected between 2011-2015. For participants who provided data through telephone interview, data collection took place between September 2011-December 2015, and for participants who provided data through in-home interview and at a data collection site, data collection took place between May 2012 and May 2015. The first follow-up was completed from 2015-2018 (Telephone interview from December 2015 to December 2018 and in-home interview and data collection site visit from July 2015 to July 2018).<br>The CLSA COVID-19 study was launched on April 15, 2020 and data were collected multiple time points over a 9-month period. The participants completed 10-minute questionnaires weekly (4 times by web) or biweekly (2 times by phone), 3 monthly questionnaires and a final 30-minute exit questionnaire between September and December of 2020.
Data exclusions	This paper includes all participant who completed the COVID-19 exit interview (n=24,114).
Non-participation	Of the 51,338 participants recruited at CLSA baseline, first follow-up was completed on 48,893 participants (95% retention) in mid-2018.<br>Of the 51,338 CLSA participants, 42,700 were invited to take part in the CLSA COVID-19 study. The 8,638 excluded individuals included those who had died (n=2,500), withdrew prior to follow-up 2 (n=3,406), required a proxy (n=318), or for administrative reasons, the most common being that current contact information was not available (n=2,414). During the recruitment process, an additional 166 participants were identified as deceased and 23 as needing a proxy to arrive at the overall eligible sample of 42,511, of which 28,559 (67.2%) agreed to participate in the CLSA COVID-19 study.
Randomization	n/a

Reporting for specific materials, systems and methods

We require information from authors about some types of materials, experimental systems and methods used in many studies. Here, indicate whether each material, system or method listed is relevant to your study. If you are not sure if a list item applies to your research, read the appropriate section before selecting a response.

Materials & experimental systems

n/a	Involved in the study
☒	☐ Antibodies
☒	☐ Eukaryotic cell lines
☒	☐ Palaeontology and archaeology
☒	☐ Animals and other organisms
☐	☒ Human research participants
☒	☐ Clinical data
☒	☐ Dual use research of concern

Methods

n/a	Involved in the study
☒	☐ ChIP-seq
☒	☐ Flow cytometry
☒	☐ MRI-based neuroimaging

Human research participants

Policy information about studies involving human research participants

Population characteristics	See above.
Recruitment	The CLSA is a national cohort. A random sample of eligible households was contacted. If an eligible individual in the household was identified, they were asked to provide their contact information to the CLSA. Individuals who responded by providing their contact information were then contacted, and those who completed all required baseline interviews and assessments and provided written informed consent were enrolled into the CLSA cohort.<br>Our study sample did not include individuals residing in the Canada's three territories, on First Nations reserves, in long-term care facilities, members of the armed forces, those who were unable to communicate in English or French, and those with severe cognitive deficits, which may have underestimated the prevalence of depressive symptoms, and limit the generalizability of our findings to community-dwelling populations only.
Ethics oversight	The study was approved by the Hamilton Integrated Research Ethics Board and by research ethics boards of all the participating institutions across Canada. Informed consent was obtained from the participants. Participants were explained the study and data collection procedures and had the opportunity to seek clarification prior to consenting.

Note that full information on the approval of the study protocol must also be provided in the manuscript.
